# 

**Published:** 1893-06

**Authors:** 


### LITERARY.

LEPROSY AND VACCINATION ; by William Tebb. London, Swan, Sonnenschein & Co. Octavo, 412 pp. Price, $1.50.

The work is dedicated to the Royal Commission on Vaccination whose protracted and patient labors have elicited the valuable evidence contained in its already published reports. The book itself is valuable for its historical research into the disease of leprosy, its increase apparently in the old world, and treatment of it. Bound in handsome cloth binding.

VAGARIES OF SANITARY SCIENCE ; by F. L. Dibble, M. D. 12 mo., pp. 462, Philadelphia, J. B. Lippincott Co. Price, $2.00.

This book, a valuable acquisition to the medical profession, to which it is dedicated, is a complete and exhaustive treatise on sanitary science, and not only should it be readily accepted in the medical circles, but in the many homes of the land. It is handsomely and substantially bound in cloth.

THE REVIEW OF REVIEWS, comparatively a new magazine, is becoming one of the best of our periodicals. Every number seems to be an improvement. The May number begins with a timely article on "The Progress of the World," appropriately illustrated. The work of the women at the Columbian Exhibition is given a liberal space, and is very happily treated. For the business man the magazine is THE periodical for his limited amount of time.

If one desires to keep up with the times in everything that is practical, "LEND A HAND," published by J. Stillman Smith & Co., Boston, is the periodical that fills in this want. "A New District," meaning upper New York, "The Singers' Alms," "Hall House," "Harvard College," "The North and Union," and other articles of interest grace the pages of the June number. Price, $2.00 per year.

THE CENTURY, for May, as usual, is a superb number. The opening chapter, "At the Fair," by W. Lewis Fraser, is an admirable descriptive story, accompanied by illustrations. "Sweet Bells Out of Tune," is still continued. Gilbert Gaul pictures life at Nicaraugua, interestingly, and many other valuable articles grace its pages.

THE BLUE AND GRAY, a thoroughly patriotic magazine, and its name suggests everything. Soldier life is depicted, its hardships, privations and other discomforts are woven into stories and these valuable additions to our nation's history, find their way to this periodical. It is a typical magazine of soldier life. Published in Philadelphia, at $2.50 per year.

THE COSMOPOLITAN leads its interesting number for May with an illustrated paper, "In the Footsteps of Dickens." Howell's continued story, "A traveler from Altruna.." "American Society in Paris," is admirably treated by Mary B. Ford. Richly illustrated is the "Omega, the last days of the World." A rich number throughout.

Our oldest society paper, the HOME JOURNAL, comes out with its usual directory of summer resorts, and it has proven a valuable feature to the paper, and largely appreciated by people of the best homes of the metropolis and elsewhere. Always bright, interesting and valuable.

The World's Fair number of the YOUTH'S COMPANION is a pleasant surprise to all who are warmly attached to that most excellent paper. Beautifully illustrated, valuable for information. Perry, Mason & Co. are to be congratulated.

THE LADIES' HOME JOURNAL, of Philadelphia, is among the best periodicals that finds its way into the homes of our people. The May number opens with a poem by Edna Dean Proctor.

